# Extranodal Natural Killer/T-cell Lymphoma With an Unusual Presentation: A Case Report and Literature Review

**DOI:** 10.7759/cureus.30734

**Published:** 2022-10-26

**Authors:** Abdulwahab Alyahya, Alhanouf Alhedaithy, Tariq Abualhamayel, Nada Aldabal, Ahmed Bukhari

**Affiliations:** 1 College of Medicine, King Faisal University, Al Ahsa, SAU; 2 Otolaryngology - Head and Neck Surgery, King Fahd Hospital of the University, Khobar, SAU; 3 Otolaryngology - Head and Neck Surgery, King Fahd Military Medical Complex, Dhahran, SAU

**Keywords:** retropharyngeal abscess, epstein-barr virus, follicular tonsillitis, extranodal natural killer/t-cell lymphoma, t-cell lymphoma

## Abstract

The Epstein-Barr virus is closely linked to a lymphoproliferative disease known as natural killer/T-cell lymphoma (NKTL). Early identification of NKTL might be challenging because it can resemble other nasopharyngeal pathologies. Contrary to the presented case, T-cell lymphoma often develops in the nasal canal and spreads to the oral cavity. Here, we present the case of a 45-year-old man with an unusual presentation of NKTL presenting initially as acute follicular tonsillitis.

## Introduction

Natural killer/T-cell lymphoma (NKTL) is an aggressive lymphoproliferative condition that is strongly associated with the Epstein-Barr virus (EBV) and accounts for 2% of all T-cell lymphomas [1,2]. NKTLs can be nodal (nNKTL) or extranodal (eNKTL), with significant clinical, pathophysiological, and genetic differences between the two types [3]. eNKTL is a rare, fast-growing (high-grade), non-Hodgkin lymphoma that grows outside the lymphatic system (hence, “extranodal”), usually in the nose. eNKTL accounts for 0.2% of all non-Hodgkin lymphomas and 1-2% of all NKTLs. Here, we present the case of a 45-year-old man with an unusual presentation of eNKTL presenting initially as acute follicular tonsillitis.

## Case presentation

**Figure 1:**
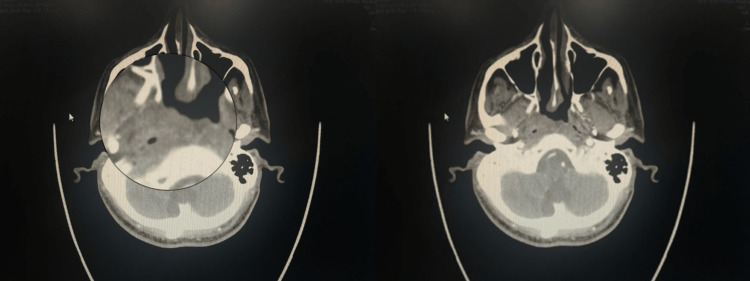
Axial computed tomography (CT) scan of the neck showing prominent asymmetry of the nasopharyngeal soft tissue, more on the right side, with no CT features suggestive of a retropharyngeal abscess.

In August 2021, a 45-year-old man with a history of acute coronary syndrome presented to the Otorhinolaryngology (ORL) Outpatient Clinic at King Fahd Military Medical Complex (KFMMC) in Al Dhahran, Saudi Arabia, with acute tonsillitis associated with subjective fever, muffled voice, and dysphagia for two weeks. Clinical examination of the intraoral cavity revealed bilateral tonsillar erythema, edema, and exudate, as well as a bulge in the right posterior pharyngeal wall without other significant findings. Urgent computed tomography (CT) scan (Figure 1) of the neck with contrast showed no signs of a retropharyngeal abscess but revealed an asymmetrical prominence of the nasopharyngeal soft tissue more on the right side associated with obliteration of the right fossa of Rosenmuller. Therefore, a CT of the chest, abdomen, and pelvis was done which showed no organomegaly or lymphadenopathy. The patient was then admitted as a case of acute follicular tonsillitis and received intravenous (IV) antibiotics and steroids and was discharged home a few days later after significant improvement in his clinical picture.

Two months later, the patient presented to the Emergency Department at KFMMC with symptoms of velopharyngeal insufficiency associated with sore throat and fever. Clinical examination of the intraoral cavity revealed congested tonsils with follicles and no other significant findings. The patient was admitted again with a diagnosis of recurrent acute follicular tonsillitis and received IV antibiotics and steroids. The patient then underwent tonsillectomy with partial uvulectomy under general anesthesia. He was discharged a few days postoperatively after significant improvement in his clinical picture.

Three weeks later, during a follow-up appointment in the ORL Clinic, the patient was clinically improving with healed tonsillar fossa and uvula. However, the histopathologic result of the excised tonsils was suggestive of T-cell lymphoma (NK/T-cell lymphoma, nasal type), and a referral to an advanced medical center for expanded immunohistochemical and molecular studies was suggested by our pathologist to confirm the diagnosis. The patient was subsequently referred urgently to a higher center, where he was admitted under hematology service for further investigation and confirmation of the diagnosis. The patient underwent multiple investigations, including a CT scan with contrast, positron emission tomography (PET) scan, virology assessment, and histopathology slides review. The PET scan showed mild activity at the site of surgery but no lymphadenopathy, organomegaly, or fluorodeoxyglucose (FDG)-avid masses. The slide review showed extensive necrosis with partial loss of CD5/CD7 with EBV infection as well as positive T-cell receptor rearrangement mimicking lymphoma. The patient’s case was discussed in their tumor board with a decision of EBV tonsillitis based on the limited and focal involvement of PET imaging, as well as complete resolution of symptoms following tonsillectomy. Therefore, the patient was referred back to us.

**Figure 2:**
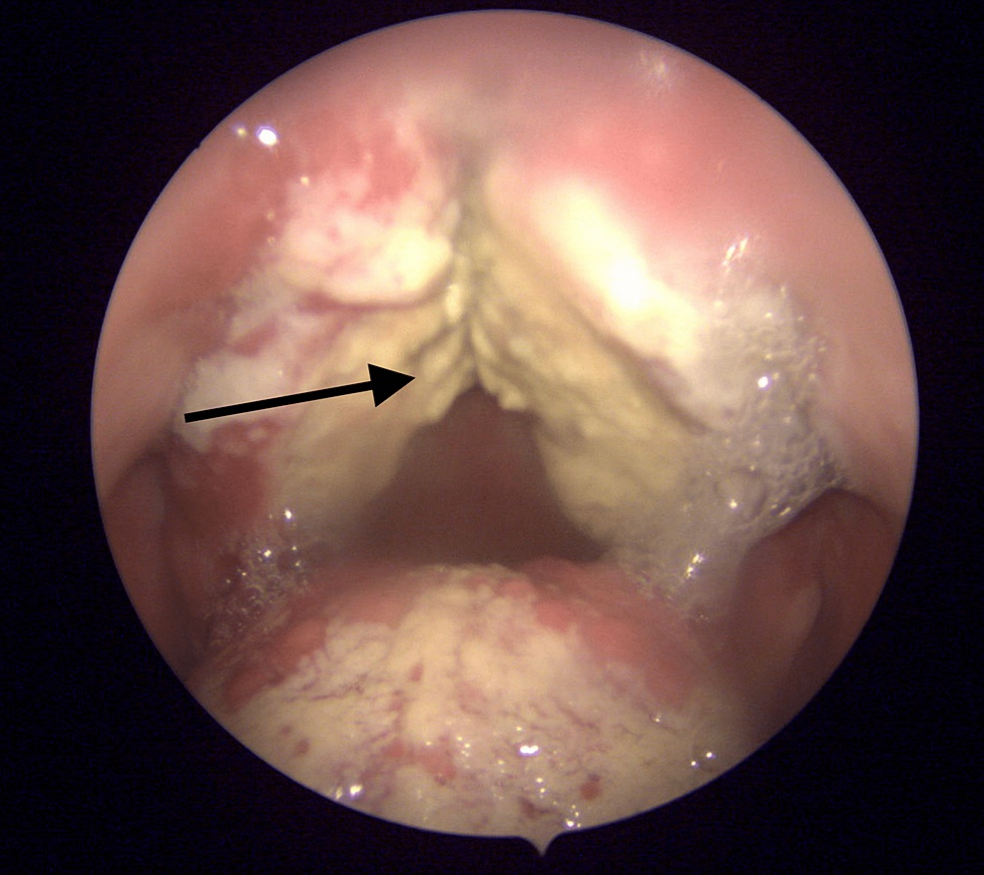
Endoscopic view of the intraoral cavity, with the arrow showing sloughed uvula, and necrotic tissue involving the soft palate.

In January 2022, during a follow-up appointment in the ORL Clinic, the patient was still complaining of sore throat, dysphagia, and mild trismus associated with worsening symptoms of velopharyngeal insufficiency, i.e., nasal regurgitation, hypernasal speech, and decreased oral intake. Additionally, he reported a weight loss of 15 kg over the past four months. Examination of the intraoral cavity revealed sloughed necrotic tissue involving the soft palate and extending laterally to involve the tonsillar pillars and extending anteriorly to involve the hard palate (Figure 2). The patient was admitted for further investigation. The infectious disease team was consulted and started the patient on tazocin and clindamycin. The patient underwent debridement of the necrotic tissue of the soft palate under general anesthesia. Nasal endoscopy and biopsy from the nasal cavity, nasopharynx, and soft palate were repeated for histopathologic examination and tissue culture. Histopathologic examination was again suggestive of T-cell lymphoma. The patient was referred to another higher center for confirmation of the diagnosis and further treatment. The patient underwent multiple investigations, including a CT scan with contrast, PET scan, bone marrow biopsy, and histopathology slides review. The PET scan showed FDG-avid activity in the nasopharynx with sub-centimetric local FDG lymphadenopathy. The bone marrow biopsy showed no evidence of bone marrow involvement by lymphoma. Histopathology slides review showed lymphoid infiltrate with focal vascular invasion, focal ulceration, and extensive coagulative necrosis. Immunohistochemical studies were performed, and the atypical cells were positive for CD56, granzyme B, and TIA1. The Ki67 proliferation index was 60%. In situ hybridization (ISH) for EBV-encoded RNA (EBER) showed reactivity in most tumor cells. The abnormal cells were negative for CD3, CD4, CD5, and CD8. Acid-fast bacilli stain and Gomori methenamine silver stain for fungal organisms were negative. Plasma DNA EBV was positive. Based on the histopathology and immunohistochemical findings a diagnosis of eNKTL was made. The patient’s case was discussed in a multidisciplinary meeting and the consensus was toward concurrent chemoradiation. The choice of chemotherapy was the SMILE (dexamethasone, methotrexate, ifosfamide, L-asparaginase, and etoposide) chemotherapy regimen. The patient is currently receiving radiochemotherapy treatment for his eNKTL in a tertiary oncology center.

## Discussion

eNKTL cell origin is rare, aggressive, and predominantly characterized by angiotropism and angiodestruction, necrosis, and association with EBV. EBV is implicated in the lymphomagenesis via clonal episomal form present prior to clonal expansion. NK cells infected by EBV secrete interleukin (IL)-9 and IL-10, which promote cell activation and proliferation [4]. Nasal presentation (around 65-80%) is the most common initial site of presentation of eNKTL; other sites can include the orbit, salivary glands, and paranasal sinuses [5,6] (Table 1). Extranasal sites (15-35%) include the gastrointestinal tract, skin, testis, lungs, spleen, and liver [7,8]. In the described case, the lymphoma began to grow in the oral cavity, which is contrary to that reported in a case series by Parker et al. and many other reported cases [9,10].

**Table 1:** An overview of the case presented in this report and other eNKTL cases reported in the literature. EBV: Epstein-Barr virus; eNKTL: extranodal natural killer/T-cell lymphoma; SMILE: dexamethasone, methotrexate, ifosfamide, L-asparaginase, and etoposide

Treatment	EBV	Complications	Site	Symptoms	Case
Tonsillectomy with partial uvulectomy + Chemo-radiotherapy	Positive	Velopharyngeal insufficiency and hard palate erosion	Tonsillar pillars and anterior to the hard palate junction	Recurrent follicular tonsillitis associated with muffled voice and B symptoms	Present case
Oral surgical debridement + chemotherapy (AspaMetDex regimen)	Positive	Oroantral fistula, and bone destruction in the affected region	Left hard palate, alveolar ridge, left second molar, and the mandibular right first molar	Expansible mass and displaying surface ulcerations	Lanzel et al. [5]
Radiotherapy + chemotherapy (DeViC regimen)	Positive	No complications were observed	Left distal portion of the soft palate and palatine tonsil crossing the midline and extending to the uvula and the oropharynx	Painful palatal lesions associated with dysphagia, nasal obstruction, and B symptoms	Andreou et al. [8]
Chemotherapy (SMILE regimen)	Positive	Inflammation and ulceration of the uvula, hard palate, palatine tonsils, nasal septum perforation, and death	The uvula, hard palate, palatine tonsils, and right upper eyelid	Chronic sinusitis associated with edema, intense pruritus of the right eyelid, and B symptoms	Lisowska et al. [12]
Chemotherapy (SMILE regimen)	Negative	Metastasis to the cervical spine associated with progressive numbness, amyosthenia, and pain in the right upper extremity	Mass in the right nasal cavity	Runny nose associated with chronic sinusitis	Pan and Luo [20]

The clinical signs and symptoms of eNKTL vary and can include nasal/upper airway obstruction, purulent discharge, epistaxis, and variable presentation of B symptoms [9]. eNKTL exhibits lesions that often present as an aggressive tumor in the nasal area, with surrounding edema, ulceration, and destruction of the nasofacial area leading to functional and esthetic deformities [10]. Lesions have also been reported to erode the hard palate, resulting in a connection between the nasal and oral cavities and giving the appearance of a lethal midline granuloma [11,12]. In this case, the patient presented initially with what appeared as simple acute tonsilitis, and his symptoms progressed to fever, weight loss, and velopharyngeal insufficiency. Additionally, erosion and ulceration of the hard palate were described in the current case, which led to a connection between the nasal and oral cavities, similar to the case reported by Kwong and Khong [11].

Diagnosis of eNKTL can be challenging because it can resemble many other differential diagnoses [12]. EBV infection should be confirmed in all cases to establish the diagnosis. Essential histologic features of eNKTL include multiple fragments of largely ulcerated tissue with extensive necrosis and diffuse infiltration of atypical lymphoid cells. Immunohistochemistry stains show the absence of surface CD4, CD5, and CD8 on the tumor cells while staining positive for cytotoxic molecules (granzyme B, perforin, and TIA-1) and CD2, CD3e, and CD56 [13]. Additionally, in situ hybridization for EBV-encoded early RNA (EBER) will be positive [14]. Routine blood counts and serum biochemistry, as well as a trephine biopsy, are required as part of the workup to diagnose eNKTL. PET/CT is currently considered the standard imaging modality for all types of lymphomas including eNKTL [15]. PET/CT is performed in newly diagnosed patients not only for proper staging but also as a prognostic indicator as these scans can be compared. It is also important to note that PET/CT standard uptake value in eNKTL is typically less than aggressive B-cell lymphomas [16]. The diagnosis of eNKTL was challenging in this case, which could have been due to loss of atypia on the initial biopsy, as well as the presence of extensive coagulative necrosis, necessitating the need for repeated biopsies for correct diagnosis. The second histopathologic results helped in confirming the malignancy and T-cell lymphoma via Immunohistochemical studies and EBER [16,17].

In the treatment of early-stage eNKTL, radiotherapy, chemotherapy, and their combination have been utilized [18]. More recently, the combination of the SMILE chemotherapy regimen is becoming the most commonly used regimen globally [19]. Novel immunotherapy drugs are currently under investigation [20]. In this case, various challenges were encountered, resulting in a four to five-month delay until a preliminary diagnosis and referral to a higher center for confirmation of the diagnosis and further management. Suspicion of eNKTL-NT is crucial for timely diagnosis to avoid diagnostic delay, especially when only highly necrotic biopsy samples are available. In addition to the medical history, clinical manifestations, and histopathologic features, immunohistochemistry and ISH confirm the diagnosis. The patient began standard treatment, which included a combination of radiation and chemotherapy in a tertiary oncology center.

## Conclusions

Because of its aggressive nature, eNKTL is uncommon but should always be considered in the differential diagnosis of such clinical manifestations. Suspicion of eNKTL is crucial for timely diagnosis, especially when only highly necrotic histological samples are apparent. To avoid complications, patients should be treated as soon as possible, which requires the engagement of multidisciplinary teams in addition to the medical history, clinical manifestations, and morphologic features, as well as immunohistochemistry to confirm the diagnosis. Furthermore, the preferred and recommended management plan is a combination of radiation and chemotherapy.

## References

[1] Ng SB, Chung TH, Kato S (2018). Epstein-Barr virus-associated primary nodal T/NK-cell lymphoma shows a distinct molecular signature and copy number changes. Haematologica.

[2] Allen PB, Lechowicz MJ (2019). Management of NK/T-cell lymphoma, nasal type. J Oncol Pract.

[3] Wang H, Fu BB, Gale RP, Liang Y (2021). NK-/T-cell lymphomas. Leukemia.

[4] Haverkos BM, Pan Z, Gru AA (2016). Extranodal NK/T cell lymphoma, nasal type (ENKTL-NT): an update on epidemiology, clinical presentation, and natural history in North American and European cases. Curr Hematol Malig Rep.

[5] Lanzel E, Syrbu SI, Hellstein JW, Stein KM, Welander S, Sousa Melo SL (2018). Destructive soft tissue mass in the maxilla/maxillary sinus. Oral Surg Oral Med Oral Pathol Oral Radiol.

[6] Takata K, Hong ME, Sitthinamsuwan P (2015). Primary cutaneous NK/T-cell lymphoma, nasal type and CD56-positive peripheral T-cell lymphoma: a cellular lineage and clinicopathologic study of 60 patients from Asia. Am J Surg Pathol.

[7] Tse E, Kwong YL (2016). Diagnosis and management of extranodal NK/T cell lymphoma nasal type. Expert Rev Hematol.

[8] Andreou A, Thermos G, Sklavounou-Andrikopoulou A (2021). Extranodal NK/T cell lymphoma, nasal type with palatal involvement: a rare case report and literature review. Head Neck Pathol.

[9] Kim SJ, Jung HA, Chuang SS (2013). Extranodal natural killer/T-cell lymphoma involving the gastrointestinal tract: analysis of clinical features and outcomes from the Asia Lymphoma Study Group. J Hematol Oncol.

[10] Parker NP, Pearlman AN, Conley DB, Kern RC, Chandra RK (2010). The dilemma of midline destructive lesions: a case series and diagnostic review. Am J Otolaryngol.

[11] Kwong YL, Khong PL (2011). Central palatal perforation in nasal natural killer cell lymphoma. Br J Haematol.

[12] Lisowska G, Zięba N, Stryjewska-Makuch G, Ścierski W, Miśkiewicz-Orczyk K, Misiołek M (2020). Natural killer (NK)//T-cell lymphoma, nasal type, with periorbital involvement: a case report and literature review. Am J Case Rep.

[13] Tse E, Kwong YL (2017). The diagnosis and management of NK/T-cell lymphomas. J Hematol Oncol.

[14] Jia Y, Byers J, Mason H, Qing X (2019). Educational case: extranodal NK/T-cell lymphoma, nasal type. Acad Pathol.

[15] Khong PL, Pang CB, Liang R, Kwong YL, Au WY (2008). Fluorine-18 fluorodeoxyglucose positron emission tomography in mature T-cell and natural killer cell malignancies. Ann Hematol.

[16] Kwong YL, Pang AW, Leung AY, Chim CS, Tse E (2014). Quantification of circulating Epstein-Barr virus DNA in NK/T-cell lymphoma treated with the SMILE protocol: diagnostic and prognostic significance. Leukemia.

[17] Gualco G, Domeny-Duarte P, Chioato L, Barber G, Natkunam Y, Bacchi CE (2011). Clinicopathologic and molecular features of 122 Brazilian cases of nodal and extranodal NK/T-cell lymphoma, nasal type, with EBV subtyping analysis. Am J Surg Pathol.

[18] Tse E, Kwong YL (2013). How I treat NK/T-cell lymphomas. Blood.

[19] Kwong YL, Kim WS, Lim ST (2012). SMILE for natural killer/T-cell lymphoma: analysis of safety and efficacy from the Asia Lymphoma Study Group. Blood.

[20] Pan Q, Luo Y (2020). Recurrence of nasal type NK/T cell lymphoma presenting as neurolymphomatosis on 18F-FDG PET/CT: a case report and literature review. Medicine (Baltimore).

